# Non-Destructive Sensor for Glucose Solution Concentration Detection Using Electromagnetic Technology

**DOI:** 10.3390/mi15060758

**Published:** 2024-06-05

**Authors:** Shasha Yang, Shiwen Gao, Yi Zhuang, Wence Hu, Junyi Zhao, Zhenxiang Yi

**Affiliations:** The Key Laboratory of MEMS, Ministry of Education, Southeast University, Nanjing 210096, China; 230228390@seu.edu.cn (S.Y.); 213220700@seu.edu.cn (S.G.); 213223805@seu.edu.cn (Y.Z.); 213212311@seu.edu.cn (W.H.); jyzhao_seu@163.com (J.Z.)

**Keywords:** blood glucose concentration, complementary split ring resonator, insertion loss

## Abstract

In this paper, a sensor using a complementary split ring resonator (CSRR) is proposed for non-destructive testing of blood glucose. By depicting the complementary split ring structure on the ground, the electromagnetic field strength between the split rings can be enhanced effectively. The structure size of the sensor by CSRR is determined by simulation, so that the insertion loss curve of the device has a resonance point at the frequency of 3.419 GHz. With a special holder created by three-dimensional (3D) printing technology, the test platform was established when the concentration of the solution varied from 0 mg/mL to 20 mg/mL. The experimental results indicate that there is an obvious linear relationship between the insertion loss *S*_21_ and the glucose concentration at the resonant frequency. Similarly, the measured real part and imaginary part of the *S*_21_ both vary with glucose concentration linearly. Based on the above experimental results, the feasibility of the sensor using a CSRR proposed in this paper for non-destructive detection of blood glucose is preliminarily verified.

## 1. Introduction

With the progress of science and technology and the development of society, great changes have taken place in people’s lifestyles and eating habits, which have sharply increased the incidence of diabetes. As a type of chronic disease, diabetes is mainly manifested in humans by abnormal glucose levels in the blood. Diabetes is a metabolic disease caused by insufficient insulin secretion or insulin utilization disorder [1,2]. Diabetes can cause a series of other diseases, and even lead to death in serious cases. Consequently, diabetic patients need to measure the changes of blood glucose levels in their body several times a day. The current medical blood glucose detection is mainly realized by taking interfinger blood and then using instruments or test papers. For patients who need to monitor blood sugar at any time, this method will cause a serious physiological and psychological burden, increasing the risk of infection. Therefore, a non-invasive, real-time monitoring technology for blood glucose is of great significance for the prevention and clinical treatment of diabetes.

Blood glucose measurement methods mainly comprise two categories: invasive and non-invasive types. The invasive blood glucose measurement method is the traditional blood collection measurement. In addition, there is also a minimally invasive blood glucose measurement method, which can effectively reduce pain in patients. On the other hand, non-invasive blood glucose measurement is painless and can be monitored in real time. In current research, non-destructive blood glucose measurement methods include infrared spectroscopy [3,4,5], photoacoustic spectroscopy [6,7], fluorescence spectroscopy [8,9,10] and other optical methods. The interactions between glucose in blood and specific wavelength of light, the absorption, scattering and transmission characteristics of light are utilized to infer the concentration of glucose in the blood [11]. In addition, biochemical methods such as reverse ion electroosmosis [12,13,14,15,16] and bioelectrical impedance [17,18] are also commonly applied for non-destructive detection of blood glucose. Moreover, the microwave method is also used to measure the concentration of glucose in blood non-invasively by microwave radiation [19]. Based on the principle of interaction between microwave and matter, this method can infer the level of glucose in blood by detecting the absorption and scattering behavior of microwave radiation in matter [20]. Previously, some sensors in the microwave field were reported to be used to measure blood glucose concentration [21,22,23,24]. However, these sensors are often used to measure blood in vitro, which cannot fully achieve the purpose of a non-destructive test. 

CSRR is a commonly used structure in microwave and radio frequency circuits, which is used to realize band resistance, filtering and coupling functions. In 2003, F. Martín et al. proposed a kind of left-handed material (LHM) with negative permeability and permittivity based on the inductive coupling of a coplanar waveguide and split-ring resonator (SRR) and metal loading with narrow metal wires [25]. In 2007, M. Navarro-Cía et al. obtained a new synthesis method of LHM by combining an electromagnetic band-gap (EBG) structure with a square split ring resonator (SSRR) in a planar microstrip circuit [26]. The synthesis of LHM can effectively promote the development of filters, couplers and other devices in traditional millimeter-wave circuit technology and the miniaturization design of millimeter-wave and microwave circuits. In 2009, Y. D. Dong et al. studied a substrate integrated waveguide (SIW) with complementary split-ring resonator (CSRR) etched on the waveguide surface, and verified the feasibility of its application based on a miniaturized waveguide filter [27]. In 2018, C. Jang et al. studied the temperature effect of glucose solution and corrected the temperature of the proposed CSRR [28]. In 2019, Palak Tripathi et al. proposed a split ring resonator (SRR) loaded on a radiation patch. The resonator has a bandwidth from 21.079 to 27.59 GHz with the sensor stacked on the tissue of patients with diabetes. The change of glucose concentration in the blood will affect the relative permittivity and conductivity of the blood, resulting in a change in the resonant frequency of the subwavelength resonator [29]. In 2020, Afia Ibnath et al. used CSRR for non-destructive detection of blood sugar levels and noted that CSRR is more suitable for this design than SRR of the same size [30].

On this basis, this study proposed a non-invasive blood glucose sensor by measuring insertion loss of the sensor using CSRR structure. A solution holder fabricated by 3D printing technology was placed over the surface of the sensor. The experiments were performed by changing the concentration of the solution in the holder. Differently from [29,30], the insertion loss was measured including the real, imaginary part and phase shift. Importantly, the trends between the insertion loss and the solution concentration are opposite at different frequency points, which is reported for the first time in relevant literature.

## 2. Theory and Simulation

### 2.1. Theoretical Analysis

Compared with other sub-wavelength resonators, such as the electric-field-coupling (ELC) [31] resonator and the complementary electric-field-coupling (CELC) [32] resonator mentioned in reference [33], the complementary split-ring resonator is only able to couple to the magnetic field or electric field perpendicular to the resonator plane, so it only acts in a specific direction; this single directional resonator is more suitable for the current application of glucose solution concentration detection. The CSRR proposed in this paper is composed of two concentric sub-wavelength SRRs which are depicted on the ground plane and placed in opposite directions, and are coupled and fed by microstrip lines. Compared with the SRR structure in which the two sub-wavelength SRRs are placed in the forward directions mentioned in reference [34], when the substrate material is unchanged, the two split-ring resonators can be placed in opposite directions to achieve a higher degree of miniaturization, which is very important for the wearability of blood glucose detection devices in the later period. When the size of the split ring structure is appropriate, the coupling between the split ring and the microstrip line can form resonance at a specific frequency [35]. As a result, the signal at that frequency is amplified, while the signal at other frequencies is suppressed. At the resonant frequency of the CSRR, the near-field electromagnetic field is strongly confined to the complementary split ring [36], so the measured object is usually placed in the complementary split ring structure. In this design, the resonant frequency can be adjusted by changing the shape and size of the split ring.

**Figure 1:**
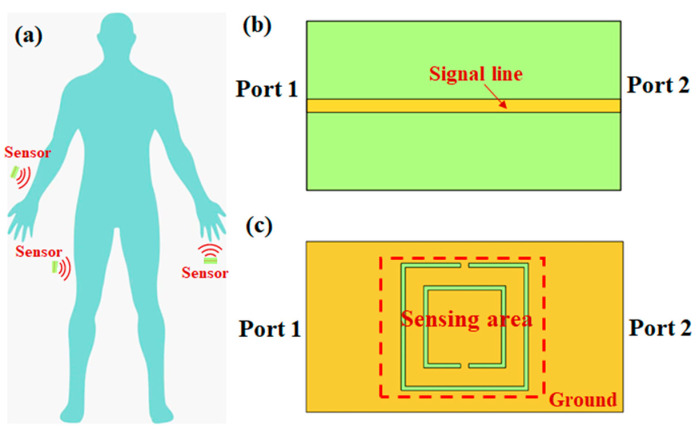
Schematic diagram of blood glucose detection: (**a**) Placement of sensors; (**b**) front surface of the sensor; (**c**) back surface of sensor based on CSRR.

In practical application, the side of the sensor engraved with a split ring structure is usually attached to the body, such as the arm, abdomen or leg, as shown in Figure 1a. A microwave signal is transmitted from Port 1 to Port 2 through the microstrip line. The change of blood glucose concentration in the human body will influence the transmission characteristics of the CSRR. Consequently, the blood glucose concentration can be predicted by detecting the change of the insertion loss.

This paper mainly analyzes the transmission characteristics of the sensor by CSRR. The insertion loss *S*_21_ is as follows:

(1) $$S_{21}=20\log_{10}{\left|\frac{V_{2}^{-}}{V_{1}^{+}}\right|}_{V_{2}^{+}=0}\left(dB\right)$$

where *V*_1_^+^ is the incident voltage of Port 1, and *V*_2_^−^ is the output voltage of Port 2 when the incident voltage of Port 2 is *V*_2_^+^ = 0. Through the construction of the equivalent circuit model of the CSRR and its theoretical analysis, the calculation formula of insertion loss *S*_21_ can be further obtained as follows:

(2) $$S_{21}=20\log_{10}\left|\frac{4Z_{0}^{2}Z_{sh}}{(jwL_{l/2}+Z_{0}+Z_{sh}){\left(Z_{in}+Z_{0}\right)}^{2}}\right|\left(dB\right)$$

where *L*_l⁄2_ is the inductance, *Z*_0_ is the port impedance, *Z_sh_* is the branch impedance and *Z_in_* is the input impedance. Obviously, the increase of glucose solution concentration will lead to the increase of branch impedance and insertion loss. As a result, based on this principle, the change of glucose solution concentration can be calculated by the insertion loss measurement for the proposed device.

### 2.2. FEM Simulation

**Figure 2:**
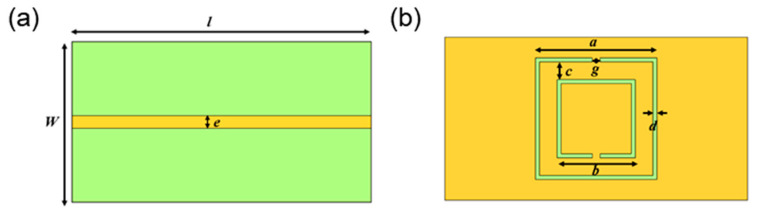
Schematic diagram of the structure size of CSRR: (**a**) front surface; (**b**) back surface.

In the design of the proposed sensor, the most important thing is to determine the sizes of the microstrip transmission line and complementary split ring. Different sizes will produce different frequency resonance points. The structure of the complementary split ring designed in this paper is square, and the structure size diagram of the CSRR is shown in Figure 2, where *a* is the length of the outer side of the split ring, *b* is the length of the outer side of the inner ring, *c* is the distance between the inner ring and the outer ring, *d* is the width of the inner ring, *g* is the width of the ring, *l* and *w* are the length and width of the substrate respectively, and *e* is the width of the microstrip line.

**Figure 3:**
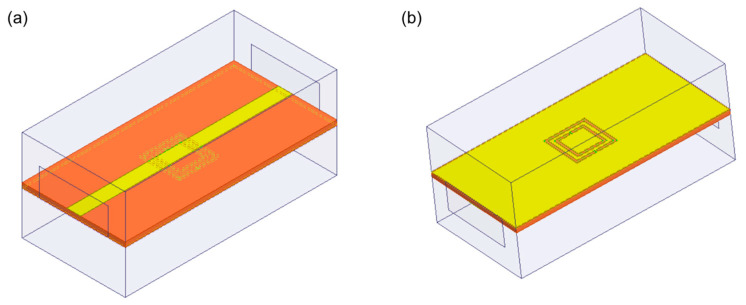
Simulation model of the sensor by CSRR in HFSS software: (**a**) front of the model; (**b**) back of the model.

**Figure 4:**
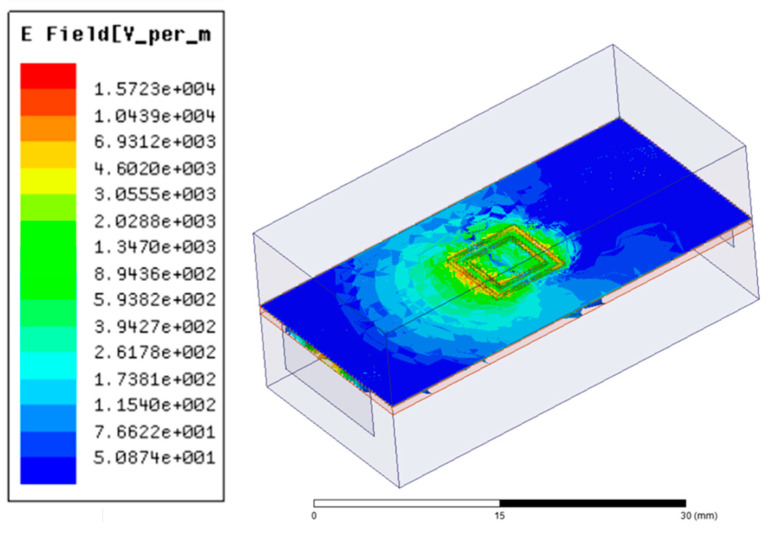
Electric field distribution in the grounding plane of the sensor by CSRR.

**Figure 5:**
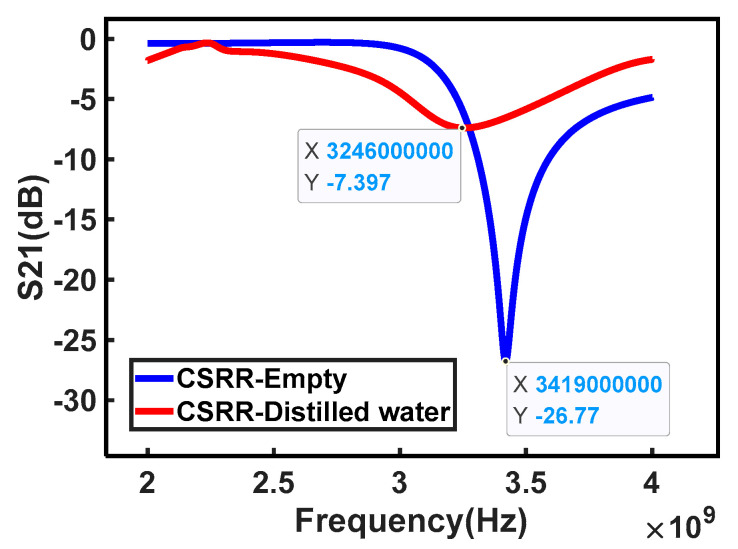
Simulation results of insertion loss *S*_21_ with and without distilled water by HFSS software.

Finite elements method (FEM) software HFSS 15.0 was utilized to simulate the microwave performance of the sensor by CSRR. Figure 3a and b show the top view and bottom view of the CSRR model in the HFSS software, respectively. Next, the insertion loss *S*_21_ of the CSRR was simulated over the frequency range of 2–4 GHz. Figure 4 shows the electric field distribution on the back of the CSRR. It can be seen that there is a strong electric field at the complementary split ring structure compared to other positions. Moreover, Figure 5 gives the simulated *S*_21_ by adding a test region to the complementary split ring structure and adding distilled water as its material, respectively. It can be seen that the insertion loss *S*_21_ of the CSRR has a resonant peak at the frequency of 3.419 GHz without the distilled water test region, while the resonant frequency shifts forward to 3.246 GHz when the test area is filled with distilled water.

**Figure 6:**
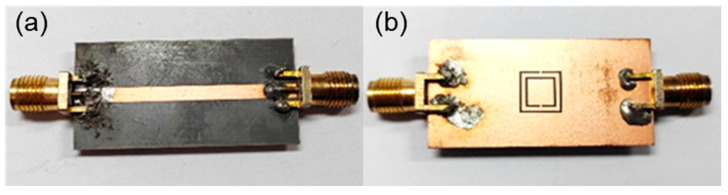
Photographs of the sensor using CSRR: (**a**) front surface; (**b**) back surface.

After optimization, the structure size of the sensor by CSRR was obtained and is shown in Table 1. The device was fabricated on RF4 substrate and Figure 6 shows photographs of the front surface and back surface of the sensor. Two SMA connectors are bonded to the sensor as the input port and output port.

## 3. Results and Discussion

**Figure 7:**
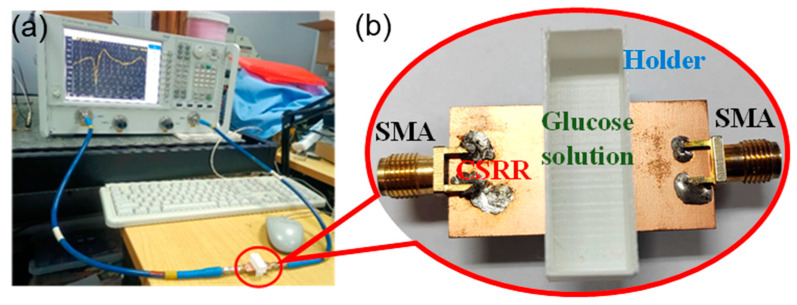
Photographs of the experimental platform: (**a**) measurement by VNA; (**b**) sample holder on the sensor.

In order to verify the feasibility of the proposed sensor using a CSRR for non-destructive detection of blood glucose, the experimental platform was established and related experiments were performed. Figure 7 shows that the vector network analyzer (VNA) was used to measure the microwave performance of the sensor with the solution in the holder. First, the two ports of the sensor using a CSRR were connected to the input/output port of the VNA via a coaxial transmission line, as shown in Figure 7a. Then, a certain amount of deionized water was added to the sample holder, and the concentration of the solution was changed by adding the same amount of glucose powder during the experiments. This method can effectively avoid the experimental errors caused by changing solutions with different concentrations. Finally, the transmission characteristics of the sensor were measured by the VNA.

**Figure 8:**
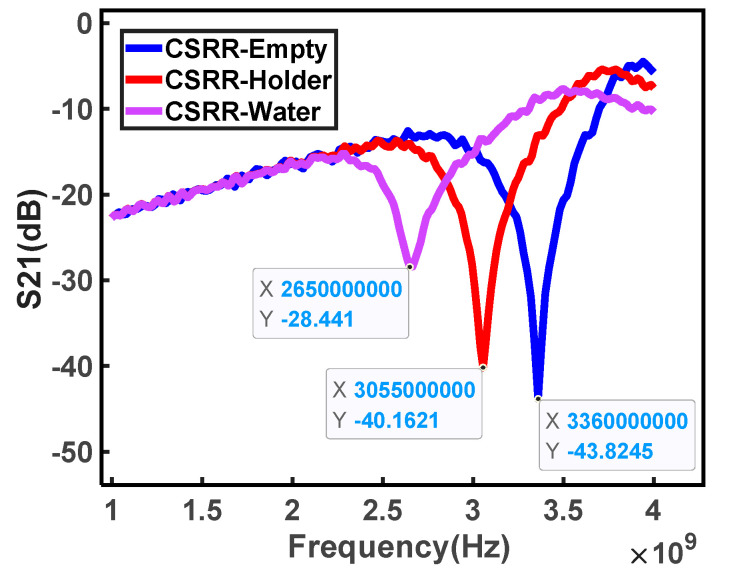
Measured insertion loss *S*_21_ of the sensor under different conditions.

Figure 8 shows the amplitude of the measured *S*_21_ for the sensor, the sensor equipped with a holder, and the sensor with a certain amount of deionized water in the holder, respectively. The measured curve indicates that the sensor has a resonance point at the frequency of 3.360 GHz, and the error is close to 0.059 GHz compared to the simulation result. The difference is caused by the fact that that the simulation was carried out under ideal conditions, and the presence of the material under test (MUT) was not considered. Additionally, the resonant frequency moved downward to 3.055 GHz when the holder was placed on the sensor. After filling the holder with water, the resonant frequency was further reduced to about 2.650 GHz.

**Figure 9:**
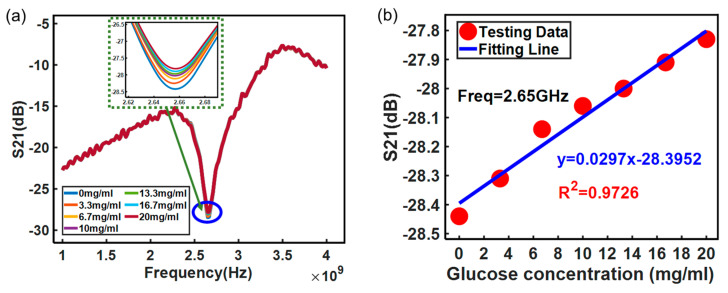
Amplitude of the measured *S*_21_: (**a**) amplitude variation curve with frequency; (**b**) amplitude variation curve with glucose concentration.

The solution concentration was varied from 0 mg/mL to 20 mg/mL and the amplitude of *S*_21_ was measured and is shown in Figure 9. Figure 9a shows that the insertion loss *S*_21_ of the sensor on which the MUT is placed will resonate at a frequency of 2.65 GHz. In addition, Figure 9b indicates that the amplitude of *S*_21_ increases from −28.45 dB to −27.80 dB linearly at the resonance frequency point when the solution concentration ranges from 0 mg/mL to 20 mg/mL. Moreover, the results are linearly fitted with a coefficient of determination of 0.9726. To demonstrate the superiority of the proposed glucose sensor, a comparison was made with the sensitivity of other microwave sensors (Table 2).

**Figure 10:**
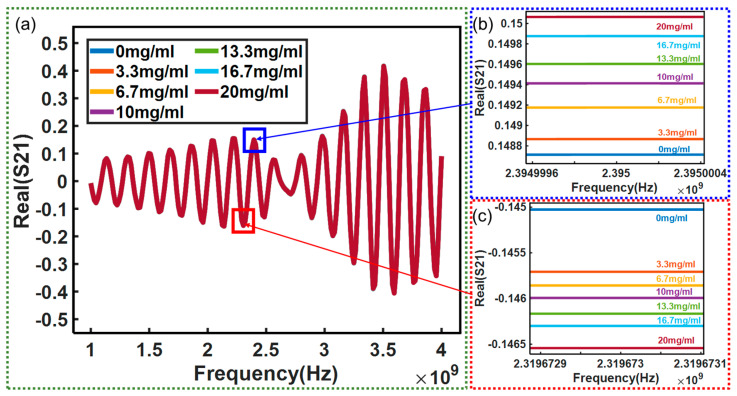
(**a**) Test results of correlation between *S*_21_ real part and frequency: (**b**) test result at 2.395 GHz; (**c**) test result at 2.319673 GHz.

**Figure 11:**
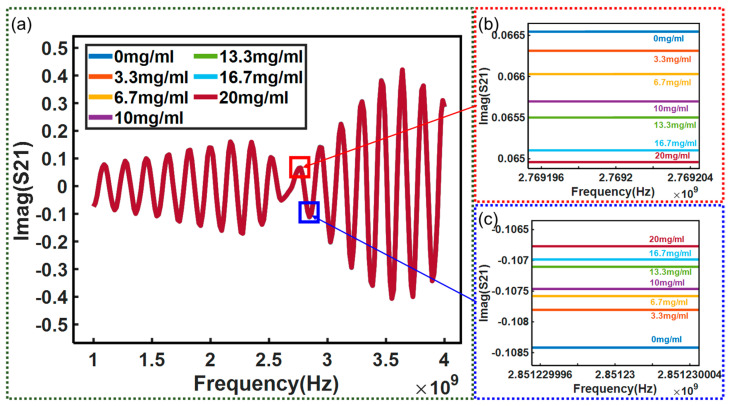
(**a**) Test results of correlation between *S*_21_ imaginary part and frequency: (**b**) test result at 2.7692 GHz; (**c**) test result at 2.85123 GHz.

**Figure 12:**
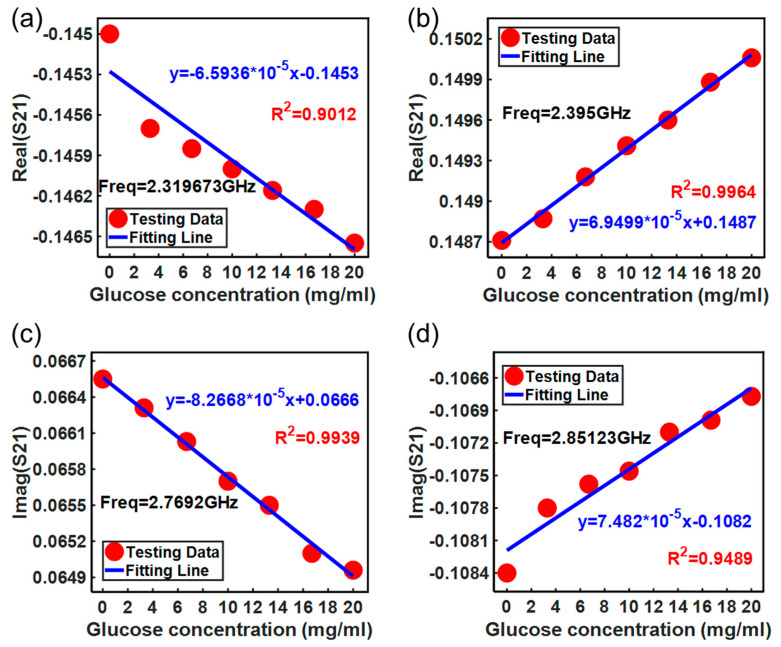
Measured real part and imaginary parts of *S*_21_ with glucose concentration: (**a**) real part test result of *S*_21_ at 2.319673 GHz; (**b**) real part test result of *S*_21_ at 2.395 GHz; (**c**) imaginary part test result of *S*_21_ at 2.7692 GHz; (**d**) imaginary part test result of *S*_21_ at 2.85123 GHz.

Moreover, the real and imaginary parts of the insertion loss *S*_21_ were also investigated. The tested results are shown in Figure 10 and Figure 11, respectively. Figure 10a and Figure 11a record the measured real and imaginary parts of the *S*_21_ over the frequency range of 1–4 GHz, respectively. In addition, local image processing was performed on the changes at specific frequency points in the real and imaginary parts of *S*_21_, as shown in Figure 10b,c and Figure 11b,c, respectively. From the figures, it can be seen more clearly that the changes in the real and imaginary parts of *S*_21_ with concentration can be observed, and the results shown in Figure 12 can be obtained. Figure 12 shows the real and imaginary parts of insertion loss *S*_21_ exhibiting a linear relationship with the glucose solution at several frequency points. However, the real part of the *S*_21_ decreases with the glucose concentration at 2.319673 GHz while it increases at 2.395 GHz. Similarly, the imaginary part of the *S*_21_ decreases with glucose concentration at 2.7692 GHz while it increases, with a coefficient of determination of 0.9489, at 2.85123 GHz. These opposite trends at different frequency points are measured and reported for the first time in this study.

**Figure 13:**
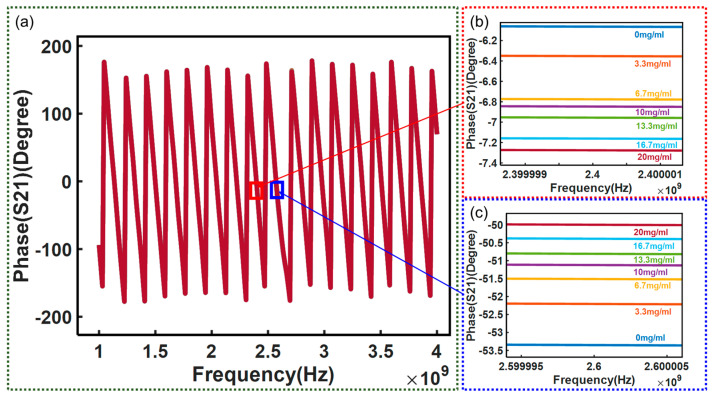
(**a**) Test results of correlation between *S*_21_ phase and frequency: (**b**) test result at 2.4 GHz; (**c**) test result at 2.6 GHz.

In addition to the amplitude, the phase shift of the *S*_21_ can also be a measure of glucose concentration. Figure 13 shows the measured phase shift of the *S*_21_ varying with the glucose concentration over the frequency band of 1–4 GHz.

**Figure 14:**
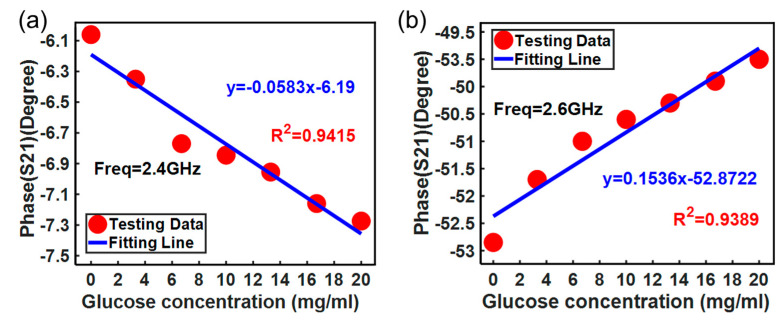
Measured phase shift variation of the *S*_21_ with glucose concentration at different frequencies: (**a**) test result at 2.4 GHz; (**b**) test result at 2.6 GHz.

The phase shift of the *S*_21_ changing with glucose solution concentration at different frequencies is also observed and plotted. Figure 14 shows that the phase shift has different linear relationships with glucose solution concentration at about 2.4 GHz and 2.6 GHz, respectively. It can be observed that the phase shift is positively correlated with glucose solution concentration at the frequency of 2.6 GHz, while it is negatively correlated, with a coefficient of determination of 0.9389, at 2.4 GHz.

The above experimental results demonstrate that the transmission characteristic of the proposed sensor using CSRR is highly sensitive to the change of glucose concentration. That is, the variation of the glucose concentration can be estimated by detecting the change of the *S*_21_ signal. However, it must be noted that there are still some potential impacts on the accuracy of current experimental results, such as the influence of the external environment, instrument errors, and possible incomplete dissolution of glucose solutions. In actual testing, these errors can only be reduced as much as possible, but it cannot be completely eliminated.

## 4. Conclusions

In this paper, a sensor with a CSRR structure is proposed, and the loop in CSRR is etched on the ground plane. The magnetic flux of the loop will make the rotating current in the loop produce its own magnetic flux, which can effectively improve the electromagnetic intensity of the near field of the sensor, and the measurement sensitivity of the sensor can be improved by placing glucose solution at the split ring structure. The structure size of proposed device was determined by HFSS simulation, and the insertion loss *S*_21_ was tested by VNA after establishing a test platform. The experimental results show that there is a linear relationship between the amplitude, real part, imaginary part and phase shift of the insertion loss *S*_21_ and the glucose concentration at a certain frequency. Above all, it is indirectly proved that, in future, a sensor using a CSRR can be applied for non-destructive detection of glucose level in human blood.

## Figures and Tables

**Table 1:** Size parameters of the proposed sensor using CSRR.

Parameter	Value (mm)
a	7
b	5.02
c	2.4
d	0.385
g	0.29
w	18
l	37
e	2.4

**Table 2:** Comparison of sensitivity of various microwave sensors for solution concentrations.

Method	Solutions to Be Tested	Sensitivity (dB/(mg/mL))
Microwave sensor [37]	Water-Sugar	0.0065
Rectangular waveguide cavity [38]	Water-Sucrose	0.018
SRR [39]	Water-glucose	0.0126 (10–50 mg/mL)
This study	Water-glucose	0.0297

## Data Availability

Data are contained within the article.
